# Real‐world analysis of hospitalizations in patients with epilepsy and treated with perampanel

**DOI:** 10.1002/epi4.12515

**Published:** 2021-08-13

**Authors:** Edward Faught, Xuan Li, Jiyoon Choi, Manoj Malhotra, Russell L. Knoth

**Affiliations:** ^1^ Department of Neurology Emory University Atlanta GA USA; ^2^ Eisai Inc. Woodcliff Lake NJ USA

**Keywords:** antiepileptic drugs, healthcare utilization, hospitalizations, pediatric epilepsy

## Abstract

**Objectives:**

(1) To evaluate risk of hospitalization following initiation of perampanel (pre‐ and post‐analysis) and (2) to compare hospitalization rates following initiation of perampanel vs lacosamide.

**Methods:**

Patients were identified from Symphony Health's Patient Integrated Database if they had a prescription for perampanel (July 1, 2014‐June 30, 2016). Patients 4‐11 years of age with any partial‐onset seizure (POS) or ≥12 years of age with any POS or primary generalized tonic‐clonic seizure (GTCS) (pre‐post); or ≥12 years of age (perampanel vs lacosamide). The first fill of perampanel (“index date”) marked the start of the analysis period. Patients had ≥1 additional fill for perampanel and ≥2 diagnoses for epilepsy or nonfebrile convulsion diagnosis during pre‐index (based on ICD‐9/ICD‐10 codes). Patients were matched using a 1:1 propensity scoring method for the perampanel vs lacosamide analysis. Primary outcome was hospitalization during the one year following medication initiation.

**Results:**

Pre‐ and post‐perampanel: N = 1771 (mean age 34 years, 55% female). One‐year all‐cause hospitalization risk ratio was 0.76 (*P* < .05) and 36.2% with hospitalization during the pre‐period vs 29.5% in the follow‐up. One‐year epilepsy‐related inpatient hospitalization risk ratio was 0.72 (*P* < .05) and 30.8% with hospitalization during the pre‐period vs 23.9% during follow‐up. In the perampanel and lacosamide cohorts, N = 1717 per cohort after matching, most baseline demographics were balanced. A higher percentage of subjects were prescribed ≥3 anti‐seizure medications for perampanel vs lacosamide (60.5% vs 57.7%, *P* < .001). The perampanel cohort had a 9.6% reduction in all‐cause hospitalizations vs 5.8% for the lacosamide cohort (*P* < .05). Epilepsy‐related hospitalizations decreased from the pre‐index rate by 9.9% for perampanel and 8.3% for lacosamide (*P* < .05). Among those with baseline hospitalizations, perampanel was associated with a 59.9% reduction in all‐cause hospitalizations vs 48.6% for lacosamide (*P* < .05), and for epilepsy‐related hospitalizations, a reduction of 65.0% vs 58.9%, respectively (*P* < .05).

**Significance:**

Perampanel was associated with a significant reduction in one‐year hospitalization risk.

**Key Points**

- Initiation of treatment with perampanel compared with the year before therapy was associated with a significant reduction in 1‐yr inpatient hospitalization risk.
- Perampanel was associated with a significantly greater reduction in hospitalizations when compared to matched patients treated with lacosamide.
- Addition of either perampanel or lacosamide reduced the one‐year risk for hospitalization.

## 1 INTRODUCTION

According to the Centers for Disease Control and Prevention, in 2015, 1.2% of the US population had active epilepsy.^1^ Children with uncontrolled epilepsy have a higher rate of hospitalization and incur greater costs than children with controlled epilepsy.^2^ Cost analyses have consistently shown higher costs, particularly related to inpatient hospitalizations, for patients with uncontrolled epilepsy when compared to controlled patients.^3^, ^4^, ^5^

New anti‐seizure medications (ASMs) may help to control seizures and reduce healthcare utilization, including hospitalizations. Perampanel (Fycompa) is a non‐competitive AMPA‐type glutamate receptor antagonist, first approved in 2012 to treat partial‐onset seizures (POS) in patients ages 12 years and older. In 2015, perampanel was approved as adjunctive treatment for primary generalized tonic‐clonic seizures (PGTCS). In 2018, the indication was extended to pediatric patients, and perampanel is now approved for the treatment of POS with or without secondarily generalized seizures in patients with epilepsy 4 years of age and older.^6^ Lacosamide (Vimpat) is a voltage‐gated sodium channel blocking agent initially approved for adjunctive therapy for POS in adults in 2009 and later approved as monotherapy in adults with POS in 2014. In 2017, lacosamide received approval for monotherapy and adjunctive therapy for the treatment of POS in patients 4 years of age and older.^7^

It is important to understand the impact of various therapies on seizure‐related outcomes. The objective of this study was twofold: The first was to evaluate the risk of inpatient hospitalization in a real‐world setting following initiation of perampanel in adult and pediatric patients diagnosed as having epilepsy. As there is the possibility that any intervention resulting in improvement could result in fewer hospitalizations,^8^ a second analysis was undertaken to compare patients who initiated therapy with perampanel to those who initiated therapy with another ASM with similar indications. Thus, the second objective was to compare hospitalization rates in patients with epilepsy following the initiation of perampanel or lacosamide.

## 2 METHODS

### 2.1 Study design and Data source

The first analysis was a retrospective pre‐ and post‐study that evaluated hospitalizations in patients with epilepsy before and after initiating treatment with perampanel. The second analysis retrospectively evaluated hospitalizations in patients with epilepsy who received perampanel and compared their outcomes with patients who received lacosamide. Lacosamide was chosen as a comparator because it has the largest branded market share in the United States as adjunctive therapy for these seizure types. Patients for both analyses were identified in Symphony Health's Patient Integrated Database if they had a prescription for perampanel filled from July 1, 2014, to June 30, 2016. This database captures adjudicated claims across the United States and includes patients with commercial, Medicare Part D, cash, assistance programs, and Medicaid coverage. The database covers approximately three‐fourths of the US population (~280 million lives) cross‐sectionally. Data are captured for approximately 70% of the retail and specialty pharmacy claims and 55% of mail order prescriptions. The database covers approximately 55% of professional medical claims and 30% of institutional claims in the country. Claims are identified based on a patient identifier associated with each clinic or retail pharmacy who contribute to the database.^9^

### 2.2 Study criteria

To be included in the pre‐ and post‐perampanel analysis, patients had to be between the ages of 4‐11 years with any partial‐onset seizure (POS) or ≥12 years of age with any POS or any primary GTCS.^10^ The first fill of perampanel was considered to be the index date. Patients were also required to have ≥1 additional fill for a prescription of perampanel following the index date fill. As the Symphony dataset does not contain eligibility information, it is difficult to determine whether treatment interruption that might be observed in the data is related to a lack of persistence, associated with patient loss to follow‐up, or due to leaving the physician's care. Thus, patients were required to have continuous clinical activity (ie, claims for physician office visits, and prescription claims) for the 1‐year period before and after the index date. Patients were required to have ≥2 diagnoses for epilepsy (ICD‐9‐CM 345.xx or ICD‐10‐CM code G40.xx) or nonfebrile convulsion diagnosis (ICD‐9‐CM code 780.39 and ICD‐10‐CM code R56.9) at least 24 hours apart during the pre‐index period.

For the perampanel vs lacosamide analysis, patients were required to be ≥12 years of age and to have had ≥1 additional fill of perampanel or lacosamide following the first fill of either agent (the first fill was considered to be the index date). Similar to the pre‐ and post‐perampanel analysis, patients were required to have continuous clinical activity for the 1‐year period prior to and following the index date. Patients were also required to have ≥2 diagnoses for epilepsy (ICD‐9‐CM 345.xx or ICD‐10‐CM code G40.xx) or nonfebrile convulsion diagnosis (ICD‐9‐CM code 780.39 and ICD‐10‐CM code R56.9) during the pre‐index period. Patients who received both perampanel and lacosamide were dropped from the analysis.

### 2.3 Outcomes

The primary outcomes of interest in the pre‐ and post‐perampanel analysis were 1‐year risk for all‐cause and for epilepsy‐related inpatient hospitalizations following initiation with perampanel therapy. Adherence information, both proportion of days covered [PDC] and medication possession ratio [MPR], were calculated as this has been shown to have because adherence has a major effect on outcomes. For the perampanel vs lacosamide analysis, the primary outcomes of interest were the all‐cause and epilepsy‐related inpatient hospitalization rates before and after treatment between the perampanel treatment cohort and the lacosamide treatment cohort.

### 2.4 Statistical analysis

#### 2.4.1 Pre‐ and post‐perampanel

Patient baseline demographic and clinical characteristics were described using mean and standard deviation for continuous variables and frequency and proportions for categorical variables. The baseline characteristics included age, gender, geographic region, year of index date, and number of prior ASMs. Comorbidities during the baseline period were those included in the Charlson comorbidity index (CCI).^11^, ^12^

The PDC was calculated by dividing the number of days in the period that were “covered” (ie, patient had medication based on medical record/claims) by the number of days in the period. The MPR was calculated by dividing the total days' supply in the period by the number of days by the number of days in the period. As this approach can overestimate adherence, both the PDC and MPR were measured. For both measures, a ratio of 0.8 or higher is considered adherent.^13^ Given these outcomes are measured from a claims database for this analysis, measures of adherence are indirectly measured and may be susceptible to artifact (ie, automatic refills are generated but never picked up by the patient).

To assess the impact of perampanel initiation, the proportions of patients who had any inpatient hospitalization for the pre‐index period and for the post‐index period were captured. The number and proportion of patients with inpatient hospitalization concordance (during both pre‐ and post‐index period) and discordance were evaluated using McNemar's test. Relative risk (RR) was calculated by dividing the proportion during the post‐index period by the proportion of the pre‐index period with 95% confidence intervals (CIs). A 95% CI for the RR lying completely above the value of 1 indicates that the risk of inpatient hospitalization is statistically significantly higher for the post‐index period than for the pre‐index period, whereas an interval below 1 indicates a lower risk of inpatient hospitalization in the post‐index period. The statistical significance analysis was performed using McNemar's test.

#### 2.4.2 Perampanel vs lacosamide

Bivariate descriptive analyses were conducted on the unmatched samples. Categorical variables were compared between the cohorts using chi‐square tests, and continuous variables were compared between the cohorts using *t*‐tests.

Propensity score matching was conducted to reduce the potential for confounding that was introduced by differences in the measured demographic and clinical characteristics between the perampanel and the lacosamide cohorts. Propensity scores were estimated using a logistic regression model, with the dependent variable being a binary indicator for cohort. Once the propensity score was estimated, patients receiving perampanel were matched to patients receiving lacosamide at a 1:1 ratio based on age, gender, geographic region, year of index date, number of prior ASMs, CCI, and evidence of previous hospitalizations. The balance in patient characteristics achieved by the propensity score matching was assessed with paired‐*t* test, McNemar's test, and standardized difference.

The 12‐month all‐cause and epilepsy‐related inpatient hospitalization rates in both the perampanel cohort and the lacosamide cohort were compared using McNemar's test. Results were presented as relative risk with corresponding 95% confidence intervals CIs.

For both analyses, all significance tests were two‐sided, and differences were considered significant at *P* < .05. All analyses were conducted using SAS 9.4 (SAS Institute).

## 3 RESULTS

### 3.1 Pre‐and post‐perampanel analysis

Of the 7363 patients identified as receiving perampanel, 1771 patients met the study criteria and were included in the pre‐ and post‐perampanel analysis (n = 119 for 4‐11 years of age with any POS; n = 1652 for ≥12 years of age with any POS or any GTCS) (Table S1). The average age was 33.6 years, 58.8% were male in the 4‐11 age‐group, and 56.5% were female in the ≥12 age‐group. Most patients in the younger age‐group were covered by Medicaid, whereas most patients in the older age‐group had commercial coverage (Table 1).

**TABLE 1:** Patient demographics and characteristics (Pre‐ and post‐perampanel)

	All (N = 1771)	Age 4‐11 years, any POS (n = 119)	Age ≥12 years, any POS or GTCS (n = 1652)
Age, years, mean (SD)	33.64 (16.84)	7.87 (2.38)	35.50 (15.88)
Median	32	8	34
Gender: Female	982 (55.4%)	49 (41.2%)	933 (56.5%)
Number of prior ASMs			
Mean (SD)	2.97 (1.37)	3.44 (1.59)	2.94 (1.34)
Median	3	3	3
Number of prior ASMs ≥3	1110 (49.5%)	86 (72.3%)	1024 (62.0%)
Comorbidity index			
Mean (SD)	0.74 (1.27)	0.84 (1.11)	0.73 (1.28%)
Median	0	0	0
Insurance plan			
Commercial	727 (41.1%)	55 (46.2%)	672 (40.7%)
Medicaid	614 (34.7%)	61 (51.3%)	553 (33.5%)
Medicare	385 (21.7%)	1 (0.8%)	384 (23.2%)
Other	45 (2.5%)	2 (1.7%)	43 (2.6%)
PDC Mean (SD)	0.65 (0.32)	0.68 (0.32)	0.65 (0.32)
Median	0.74	0.82	0.74
PDC ≥0.8	839 (47.4%)	66 (55.5%)	773 (46.8%)
MPR Mean (SD)	0.67 (0.33)	0.69 (0.33)	0.67 (0.33)
Median	0.78	0.83	0.76
MPR ≥80%	877 (49.5%)	67 (56.3%)	810 (49.0%)
Discontinuation			
Yes	1038 (58.6%)	65 (54.6%)	973 (58.9%)
Censored	733 (41.4%)	54 (45.4%)	679 (41.1%)
Days on therapy			
Mean	244	253	241
Median (95% CI)	306 (283, 331)	349 (268, n/a)	295 (270, 324)

Abbreviations: ASM, anti‐seizure medication; CI, confidence interval; GTCS, generalized tonic‐clonic seizure; MPR, medication possession ratio; PDC, proportion of days covered; POS, partial‐onset seizure; SD, standard deviation.

Table 1 also shows adherence in the two groups. Overall, slightly less than half (47%) had a PDC ≥0.8, whereas almost half of all patients had an MPR ≥80%. Patients in the younger cohort were slightly more adherent from both a PDC and MPR perspective. Patients in the younger age‐group had a longer mediation duration of therapy and a lower percentage of patients discontinuing therapy at the end of the analysis (Table 1).

**FIGURE 1:**
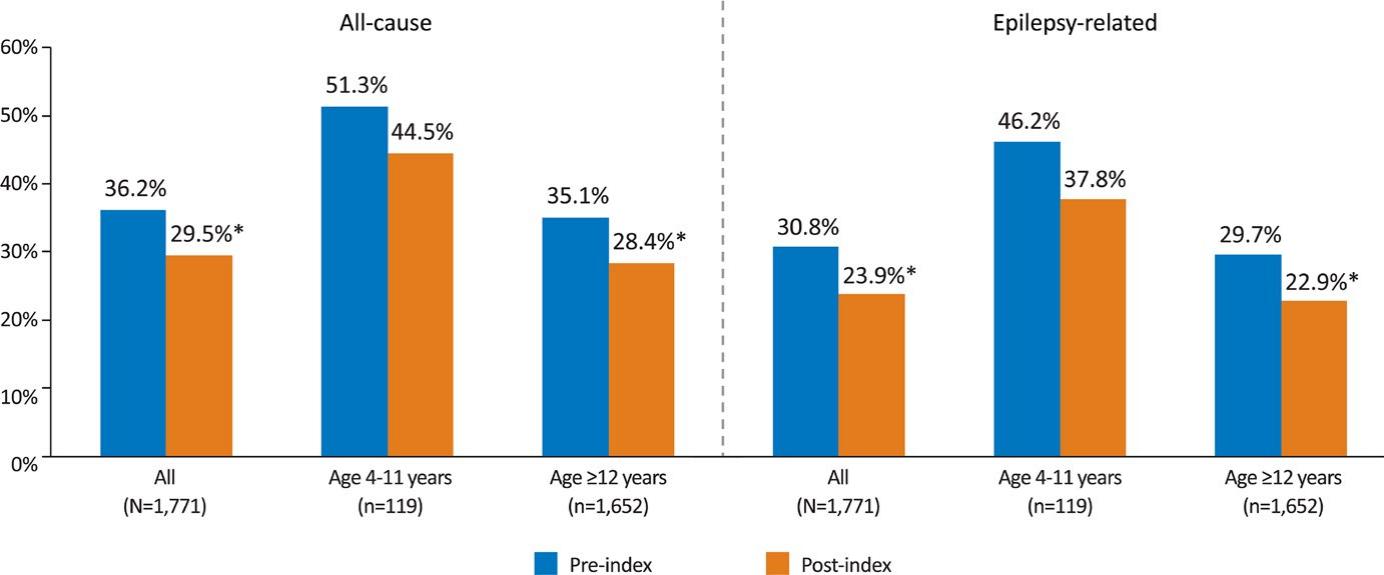
All‐cause and epilepsy‐related hospitalization rates pre‐ and post‐perampanel therapy. *P < .05

All‐cause rates of inpatient hospitalization (regardless of adherence status) were lower after initiation of perampanel therapy, as were epilepsy‐related hospitalization rates (Figure 1).

**FIGURE 2:**
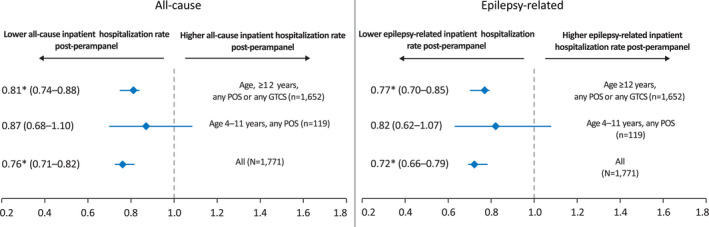
Risk ratio of all‐cause and epilepsy‐related inpatient hospitalization. *P < .05. GTCS, generalized tonic‐clonic seizure; POS, partial‐onset seizure

Risk for hospitalization was significantly lower overall and in the ≥12 years of age‐group. Risk was lower in the 4‐11 years of age‐group, but did not reach significance. Results were similar for all‐cause hospitalization and epilepsy‐related hospitalization (Figure 2).

### 3.2 Perampanel vs lacosamide

In the second analysis, 7363 patients were identified initially for receiving a perampanel prescription, and 121 705 patients were identified as receiving a lacosamide prescription. After inclusion and exclusion criteria were applied, a total of 3434 patients were matched after PSM, with 1717 patients included in each treatment cohort (Table S2). Baseline demographics and clinical characteristics were similar across cohorts, with nearly half of patients initiating perampanel or lacosamide in 2015. The median number of prior ASMs was 3. Table 2 shows additional characteristics among the matched study cohort.

**TABLE 2:** Propensity score‐matched demographic characteristics of the study cohort (Perampanel vs Lacosamide)

Characteristic	Perampanel (n = 1717)	Lacosamide (n = 1717)	Standardized difference (Matched sample)	Standardized difference (Original unmatched sample)
Age, Years, Mean [Median] (SD)	35.5 [33] (16.13)	35.3 [33] (15.51)	0.015	0.518
Gender, Female, n (%)	956 (55.7%)	958 (55.8%)	0.002	0.016
Year of Index, n (%)				
2014	418 (24.3%)	414 (24.1%)	0.0019	0.088
2015	856 (49.9%)	872 (50.8%)		
2016	443 (25.8%)	431 (25.1%)		
Number of prior ASMs, Mean [Median] (SD)	2.88 [3] (1.30)	2.83 [3] (1.32)	0.036	0.518
Number of prior ASMs ≥3, Yes, n (%)	1039 (60.5%)	990 (57.7%)	0.058	0.827
Comorbidity Index, Mean [Median] (SD)	0.65 [0] (1.15)	0.60 [0] (1.12)	0.043	0.439
Pre‐index overall inpatient hospitalization, Yes	599 (34.9%)	619 (36.1%)	0.024	0.337

**FIGURE 3:**
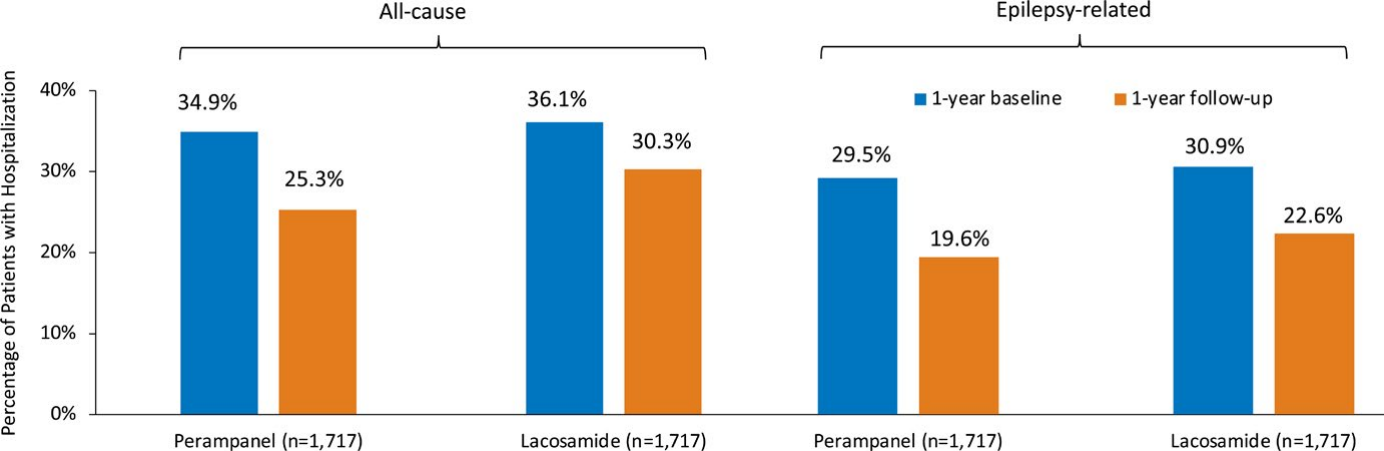
Inpatient hospitalization during baseline and follow‐up

A reduction in all‐cause and epilepsy‐related inpatients hospitalizations in the follow‐up period was observed across both treatment cohorts. During the follow‐up period, all‐cause inpatient hospitalization percentages were significantly lower for patients initiating perampanel compared to lacosamide (25.3% vs 30.3%; *P* < .05). This trend held for epilepsy‐related hospitalizations (19.6% and 22.6% for perampanel and lacosamide, respectively, *P* < .05) (Figure 3). The relative risk (RR) of 12‐month all‐cause inpatient hospitalization for perampanel patients vs lacosamide patients was 0.83 (95% CI: 0.75, 0.93) and for epilepsy‐related hospitalization was 0.87 (95% CI: 0.76, 0.99).

**FIGURE 4:**
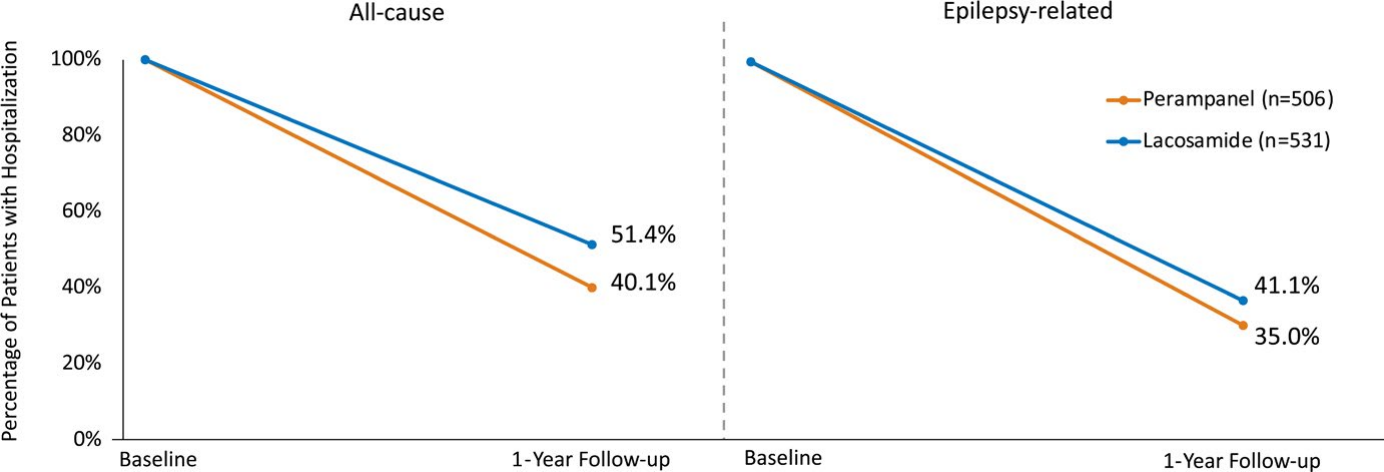
Inpatient hospitalization for patients who had any inpatient hospitalization in the baseline

Among patients (n = 1037) having at least 1 inpatient hospitalization during the baseline period, the percent of patients with an all‐cause hospitalization in the follow‐up period was significantly lower among patients receiving perampanel compared with lacosamide (40.1% vs 51.4%; *P* < .05 [Figure 4]). Similarly, the percentage of patients with an epilepsy‐related hospitalization was significantly lower in the perampanel cohort compared to the lacosamide cohort (35.0% vs 41.1%; *P* < .05 [Figure 4]).

## 4 DISCUSSION

A decrease in hospitalization rates was observed after therapy with perampanel was initiated. The risk for hospitalization in the entire cohort decreased, but not significantly among the 4‐11 age‐group. The sample size was notably smaller for this group, which may explain the lack of significance. Patients who are younger than 18 years of age and older than 65 years of age are known to have higher rates of hospitalization.^14^ Thus, these results may reflect an artifact of disease severity at different ages. Results of the pre‐ and post‐perampanel analysis indicated that children aged 4‐11 years had better adherence than the older age‐groups. Furthermore, more than half of patients in each age cohort (54.6% and 58.9% for the age 4‐11 and ≥12 cohorts, respectively) discontinued therapy by the end of the year after perampanel initiation. Though this analysis did not explore reasons for discontinuation, it is important to note that adherence rates may impact therapy success and outcomes including hospitalization.

A previous pre‐ and post‐analysis of 2508 adult patients receiving perampanel therapy indicated that there were significantly lower rates of all healthcare resource utilization than in the pre‐treatment period, including hospitalizations and outpatient visits.^15^ Results from this study are congruent with our analysis.

Both perampanel and lacosamide reduced all‐cause and epilepsy‐related hospitalizations, but the percentage of patients with an all‐cause hospitalization was significantly lower for patients who received perampanel. Patients who had at least one hospitalization in the baseline and were treated with perampanel had a significantly lower proportion of all‐cause or epilepsy‐related hospitalizations. As the average age in the second analysis was 35.5 years for perampanel and 35.3 years for lacosamide (inclusion criteria age ≥12 years), the impact of these treatments on children was not measured.

Children <12 years of age with uncontrolled epilepsy have more hospitalizations (31% vs 12%) and incur greater overall ($30 343 vs $18 206) and epilepsy‐related costs ($16 894 vs $7979) (all *P* < .001) in comparison with children with well‐controlled epilepsy in a study by Cramer, et al In this study, epilepsy accounted for half of overall costs, with comorbid conditions contributing to additional healthcare utilization and costs. Children in the study who had uncontrolled epilepsy also had a higher CCI.^2^ In our analysis, the 4‐11 age‐group also had a higher CCI (0.84) compared with the ≥12 years of age cohort (mean CCI of 0.73).

There are limitations for our study. Symphony Health is a transaction‐based data source and may not contain all patient‐level claims for each subject due to claim adjudication through other networks. Also, claims databases are designed for administrative usage and may contain errors or omissions in codes for procedures, diagnoses, or dispensing. As this is claims data, it is not possible to determine why some patients would be prescribed one ASM over another, and no direct assessment of patient adherence is possible. The analysis was conducted based on patients with a prescription for perampanel or lacosamide in combination with a diagnosis code for seizures or epilepsy. The analysis did not examine the dosage levels for perampanel or lacosamide. Administrative claims data are unreliable for determining seizure type or epilepsy syndrome beyond the broad categories of POS and PGTCS. We have used the seizure terminology current at the time of the study, but we note that the 2017 International League Against Epilepsy classification refers to POS as “focal seizures (with or without spread to tonic‐clonic) and to PGTCs as ‘bilateral tonic‐clonic seizures, presumed genetic etiology”.^15^, ^16^ Symphony is a provider‐based data source, and so patients can appear potentially as multiple persons when seen by different doctors, although Symphony uses an algorithm to minimize this issue. Clinical activity was used as a proxy for eligibility, a technique which may not have captured all eligible patients. Furthermore, the impact of perampanel usage on emergency department visits could not be examined, as these were not identified separately in the database and may be included under either outpatient or inpatient visits. Variables such as seizure control, quality of life, and medication side effects are not captured in claims data and thus are beyond the scope of this study. In the perampanel vs lacosamide analysis, treatment groups were highly imbalanced before propensity score matching. Prior to matching, the mean age in the perampanel cohort was 33, compared with 45 in the lacosamide cohort. Patients in the perampanel had a higher number of prior ASMs (3 vs 2) and a higher percentage of patients who had received ≥3 ASMs (60.9% for perampanel vs 23.2% for lacosamide). Without this technique, fewer than 20% of lacosamide patients matched adequately with perampanel patients. As a result, the findings from this study may not be generalizable to all lacosamide patients.

The reason that perampanel treatment was slightly more effective than lacosamide treatment in reducing hospitalizations is unclear. Perampanel's mechanism of action is unique among currently available ASMs, whereas the primary mechanism of action of lacosamide is shared by many ASMs. While it is true that lacosamide's mechanism is not exactly identical to that of other sodium‐channel blocking drugs, it is possible that patients relatively resistant to the effects of this class of drugs might benefit from use of a drug with a different action.^17^

## 5 CONCLUSION

In patients with epilepsy, initiation of treatment with perampanel, when compared with 1‐year clinical activity prior to therapy initiation, was associated with a significant reduction in 1‐yr all‐cause and epilepsy‐related inpatient hospitalization risk. Treatment with perampanel was associated with a significantly greater reduction in all‐cause and epilepsy‐related hospitalizations when compared to matched patients treated with lacosamide. Both drugs used as adjunctive ASMs for these cohorts were effective in reducing hospitalizations.

## CONFLICT OF INTEREST

EF has served as a consultant for Eisai, Biogen, UCB Pharma, SK Life Science, and Supernus, has received research support from UCB Pharma, and is on the editorial boards of the Annals of Neurology and Epilepsy Currents. All other authors were employees of Eisai, Inc, at the time this study was conducted. We confirm that we have read the Journal's position on issues involved in ethical publication and affirm that this report is consistent with those guidelines.

## Supporting information

Table S1‐S2
