# Supplementary material for: Real‐world analysis of hospitalizations in patients with epilepsy and treated with perampanel
Source: Epilepsia Open. 2021 Aug 13;6(4):645–52. doi: 10.1002/epi4.12515 (PMC8633480; doi:10.1002/epi4.12515)
Supplement: Supplementary file 1 — Table S1‐S2 [file EPI4-6-645-s001.docx]

**Table S1. Study Population Attrition Table Pre-Post Perampanel Analysis**

| **Inclusion criteria** | **Patients Remaining (N)** |  |
| --- | --- | --- |
| Patients who were taking perampanel (≥ 1 prescription) during the identification period from 07/01/2014 to 06/30/2016. The first prescription date was defined as index date. Patients were also required to have at least another prescription after index one. | 7,363 |  |
|  |  |  |
|  |  |  |
| Patients who had pre-index 12-month and post-index 12-month continuous clinical activity | 5,825 |  |
|  |  |  |
|  |  |  |
| Patients who had ≥2 medical diagnoses of epilepsy or convulsions during pre-index | 2,918 |  |
|  |  |  |
|  |  |  |
| Patients age 4 years and older | 2,880 |  |
|  |  |  |
|  |  |  |
| Patients who had no previous perampanel use | 2,495 |  |
|  |  |  |
|  |  |  |
| Patients were selected basing on the recent updated perampanel indications: (a) Age 4-11 with any POS (b) Age 12+ with any POS or any GTCS | **1,771** |  |
|  |  |  |
|  |  |  |

**Table S2: Study Population Attrition Table Perampanel vs Lacosamide**

| **Inclusion criteria** | **Patients Remaining in the Parampanel Cohort** | **Patients Remaining in the Lacosamide**  **Cohort** |  |
| --- | --- | --- | --- |
| Patients who were taking perampanel / lacosamide (≥ 1 prescription) during the identification period from 07/01/2014 to 06/30/2016. The first prescription date was defined as index date. Patients were also required to have at least another prescription after index one | 7,363 | 121,795 |  |
|  |  |  |  |
|  |  |  |  |
| Patients who had pre-index 12-month and post-index 12-month continuous clinical activity | 5,771 | 76,838 |  |
|  |  |  |  |
|  |  |  |  |
| Patients who had ≥2 medical diagnoses of epilepsy or convulsions during pre-index | 2,496 | 29,718 |  |
|  |  |  |  |
|  |  |  |  |
| Patients age ≥12 years old | 2,880 | 27,762 |  |
|  |  |  |  |
|  |  |  |  |
| Patients who had no previous perampanel / lacosamide use | 2,495 | 13,645 |  |
|  |  |  |  |
|  |  |  |  |
| Patients who were not prescribed lacosamide / perampanel during the identification period (i.e. perampanel patients could not have received lacosamide and vice versa) | 1,736 | 13,462 |  |
|  |  |  |  |
|  |  |  |  |
| **1:1 Propensity Score Matching** | **3,434** | |  |
